# Chromogenic Bacteria of the Human Mouth

**Published:** 1888-08

**Authors:** W. D. Miller

**Affiliations:** Berlin, Germany


### Independent Practitioner.

Vol. IX. August, 1888. No. 8.

Note.—No paper published or to be published in another journal will be accepted for this department. All papers must be in the hands of the Editor before the first day of the month preceding that in which they are expected to appear. Extra copies will be furnished to each contributor of an accepted original article, and reprints, in pamphlet form, may be had at the cost of the paper, press-work and binding, if ordered when the manuscript is forwarded. The Editor and Publishers are not responsible for the opinions expressed by contributors. The journal is issued promptly. on the first day of each month.

### Original Communications.

### CHROMOGENIC BACTERIA OF THE HUMAN MOUTH. — THEIR RELATION TO THE DIFFERENT COLORS OF DECAYED DENTINE.

BY PROF. W. D. MILLER, BERLIN, GERMANY.

I need scarcely remark that many theories have been proposed to account for the various colors presented by decayed dentine. The best known is that of Watt, which attributes these colors to the action of various mineral acids, supposed to be concerned in the production of caries. Another view assigns the chief role to articles of food, drink, etc., which do certainly sometimes produce discoloration of the decayed as well as of the healthy tooth tissue. A third would have us believe that the color comes from within, and is one of the results of the vital action of the tooth itself. A fourth theory would hold the chromogenic bacteria, micrococcus prodigiosus, etc., accountable for the various colors found.

Chromogenic bacteria are found by no means seldom in the human mouth; being widely distributed in nature it naturally happens that they find their way into the oral cavity. A plate of gelatine exposed to impure air for a short time almost invariably develops one or more colored colonies; green, various shades of yellow, brown, and red, often being represented on one plate.

As a rule, the colorless bacteria predominate in the human mouth to such an extent that the chromogenic bacteria, if present, cannot be detected; occasionally, however, they may be easily recognized even by the naked eye. I have observed a brick-red color more frequently than any other, on the lingual surface of the lower front teeth and on the buccal surface of the molar teeth. A culture of this bacterium is seen in fig. 3 [1]. The cultivation succeeds with considerable difficulty, and I have been able only once to obtain a pure growth from the deposit taken from the teeth.

A number of times I have found cavities of decay, usually dark brown, in which the surface of the dentine was colored with a bright yellow mass of cheesy consistency. I have not been able to obtain a pure culture of this bacterium on gelatine, though I have repeatedly observed a slight growth on potatoes.

Any one who will keep a lookout for the two appearances just described will certainly see them. A case which was interesting in more than one respect occurred at the polyclinic of the Dental Institute at Berlin, a few weeks ago. A man presented himself, having a swelling on the right side of the face, almost as large as a man's fist, connected with the inferior wisdom-tooth (impeded eruption). The larger portion of the surface of the mouth and tongue was covered with a canary yellow deposit, which could not be accounted for by anything that the patient had taken into his mouth.

I found a bacillus in the pus which was evacuated upon the extraction of the tooth, and also in the yellow layer upon the surface of the cheeks and gums, which reproduced the same color in pure cultures and besides showed considerable pathogenic action. I had no opportunity to see the patient again, consequently do not know the result of the infection. A pure culture of this micro-organism on gelatine is seen in fig. 4 of the plate.

---

1 See lithographic plate.

I have found in the human mouth and isolated no less than eight different kinds of bacteria which produce a yellow pigment, not including the well-known yellow sarcina. These bacteria are themselves yellow, but do not impart any color to the culture medium.

I have made a great many attempts to cultivate the supposed bacterium of greenstain, but so far without success. I have indeed isolated five different bacteria from the mouth which *impart a green color to the culture media,* although themselves colorless, and all of which grow well on the usual media, but I do not bring any of them into causal connection with the greenstain, since, as far as my observation goes, the bacterium of greenstain, if there be such a thing, does not grow on gelatine. I found one of these in the contents of an alveolar abscess; it grew with tolerable rapidity, liquefying the gelatine (see fig. 1). If cultivated without the presence of oxygen no color is developed, but if the culture is shaken with air, it will, in a few seconds, assume a beautiful green color. I found the second in a cavity of decay, and the other three in my search for the supposed bacterium of pyorrhœa alveolaris; they are colorless, but impart a beautiful opalescent color to the gelatine, one of them having, at the beginning, a decidedly bluish tinge. A pure culture of one of these is seen in fig. 2. It is, however, impossible to reproduce the opalescent character in the figure. The cultures of one bacterium obtained from the mouth have a red color on the surface, but are colorless beneath the surface (fig. 3); the protoplasm of the living cells contains the coloring matter, and no color is imparted to the gelatine. Another has a reddish color also confined to the bacteria themelves. Cultures of still another have a decided brownish color (fig. 5). It liquefies the gelatine, and sinks to the bottom as a brownish irregular mass.

I shall not enter into a discussion of the biology of these bacteria. At present we are interested in the question as to what part they may take, if any, in the production of the various colors or shades of color in carious dentine. I stated my view as to the part played by chromogenic fungi in pigmenting carious dentine in the INDEPENDENT PRACTITIONER for 1884. If the question is asked whether any of the many chromogenic bacteria referred to in the article are concerned in the process, we must answer no; at least not more than the many other bacteria in the human mouth. The green-producing bacteria, which I have named *Bacteria viridantia,* are excluded, because green dentine does not occur. Those which assume a yellow color (*Bacteria flavescentia*) cannot be looked upon as the direct cause of the yellow shade of carious dentine, because the color is confined to the micro-organisms themselves, the medium on which they are cultivated becoming very little if at all stained. In the case of dentine, the relations are exactly the opposite; the dentine becomes stained while the micro-organisms remain colorless (white). This is probably well known to those who made a few sections of carious dentine. The coloring of carious dentine is, in my opinion, explained in an altogether different manner. The following description, the different parts of which are illustrated in figs. 6–9 inclusive, was taken from actual experiments. I inoculated a tube of culture gelatine with a bacterium obtained from carious dentine; almost any bacterium which liquefies the gelatine would, however, have served the same purpose. In about two weeks the gelatine was completely melted and a white mass of bacteria lay on the bottom of the tube.

At the beginning of the experiment the gelatine had only the slight yellowish tinge often present in culture gelatine (see fig. 6); gradually, however, a brownish color made its appearance, giving the tube, at the end of four weeks, the color seen in fig. 7. After ten weeks it appeared as in fig. 8, the color gradually growing darker, long after all the bacteria were dead. An old dry culture, 6–8 months old, presented the color of fig. 9, or about that of the black spots (so called caries nigra) often seen on the approximal surfaces of teeth where caries once began and then ceased after the removal of the approximating tooth. Organic matter undergoing decomposition assumes, as is well known, a dark color, and the same is true of decaying dentine. The colors characteristic of decaying dentine do not exist in the very beginning of the decay, but appear subsequently. The more recent or acute the decay the less the discoloration; the older or more chronic the decay the deeper the color.

There is, however, another factor which may play a part in the discoloration of dentine, more particularly in those that contain dead pulps; the latter sometimes become intensely black, and it is to these in particular that the following suggestion refers. My attention has been recently called to the fact that iron had by recent experiments been found in a variety of tissues where it had not previously been detected. This discovery led to the thought that iron might be present in the dental pulp, and that in such case the black color of putrid pulps might be accounted for by the formation of the sulphide of iron. I made a few preliminary experiments relating to this question, the results of which I here give. The tests were made in the following manner. A tooth was cracked in a porcelain mortar, so as to thoroughly expose the pulp, and then placed in a mixture of dilute hydrochloric acid, to which was added a small proportion of a 10 per cent. solution of ferro-cyanide of potassium. The hydrochloric acid, as well as the water used for diluting it, must be free from iron. Neither must any iron instrument be brought in contact with the freshly broken surfaces of the tooth. Those parts of the tooth containing iron, even in minute quantities, will, after an exposure of from one to sixty minutes, assume a blue color—Prussian blue being formed. One source of error is introduced in the necessary use of an iron instrument in extracting the tooth, but this will only affect those points on the external surface of the tooth with which the forceps come in contact, and may therefore be easily eliminated.

I have found iron 1) constantly in Nasmyth's membrane (probably only as a deposit from external sources); 2) in the dental pulp, though not constantly; 3) in carious dentine almost constantly, sometimes a bright blue line forming on the border between the decalcified and normal tissue, a rather remarkable appearance for which I can, at present, attempt no explanation; 4) in enamel, particularly around the margin of cavities of decay, also sometimes in decalcified enamel. [1]

It seems, consequently, not impossible that the sulphide of iron which would be formed during putrefaction of the pulp may have something to do with the discoloration of the same. Whether sulphide of iron may be formed through decay of the dentine or enamel in sufficient quantity to aid in discoloring the same, I cannot say; at present I doubt it. Further experiments may furnish an answer to this question.

---

1 Traces of iron have been detected (as is well known) by chemical analysis in both dentine and enamel.

